# Expression profile of the *N-myc Downstream Regulated Gene 2 *(*NDRG2*) in human cancers with focus on breast cancer

**DOI:** 10.1186/1471-2407-11-14

**Published:** 2011-01-12

**Authors:** Anders Lorentzen, Rikke H Lewinsky, Jette Bornholdt, Lotte K Vogel, Cathy Mitchelmore

**Affiliations:** 1 Eucaryotic Cell Biology Research Group, Department of Science, Roskilde University, Roskilde, Denmark; 2 Department of Cellular and Molecular Medicine, University of Copenhagen, Blegdamsvej 3, Denmark

## Abstract

**Background:**

Several studies have shown that *NDRG2 *mRNA is down-regulated or undetectable in various human cancers and cancer cell-lines. Although the function of *NDRG2 *is currently unknown, high *NDRG2 *expression correlates with improved prognosis in high-grade gliomas, gastric cancer and hepatocellular carcinomas. Furthermore, *in vitro *studies have revealed that over-expression of NDRG2 in cell-lines causes a significant reduction in their growth. The aim of this study was to examine levels of *NDRG2 *mRNA in several human cancers, with focus on breast cancer, by examining affected and normal tissue.

**Methods:**

By labelling a human Cancer Profiling Array with a radioactive probe against *NDRG2*, we evaluated the level of *NDRG2 *mRNA in 154 paired normal and tumor samples encompassing 19 different human cancers. Furthermore, we used quantitative real-time RT-PCR to quantify the levels of *NDRG2 *and *MYC *mRNA in thyroid gland cancer and breast cancer, using a distinct set of normal and tumor samples.

**Results:**

From the Cancer Profiling Array, we saw that the level of *NDRG2 *mRNA was reduced by at least 2-fold in almost a third of the tumor samples, compared to the normal counterpart, and we observed a marked decreased level in colon, cervix, thyroid gland and testis. However, a Benjamini-Hochberg correction showed that none of the tissues showed a significant reduction in *NDRG2 *mRNA expression in tumor tissue compared to normal tissue. Using quantitative RT-PCR, we observed a significant reduction in the level of *NDRG2 *mRNA in a distinct set of tumor samples from both thyroid gland cancer (p = 0.02) and breast cancer (p = 0.004), compared with normal tissue. *MYC *mRNA was not significantly altered in breast cancer or in thyroid gland cancer, compared with normal tissue. In thyroid gland, no correlation was found between *MYC *and *NDRG2 *mRNA levels, but in breast tissue we found a weakly significant correlation with a positive r-value in both normal and tumor tissues, suggesting that *MYC *and *NDRG2 *mRNA are regulated together.

**Conclusion:**

Expression of *NDRG2 *mRNA is reduced in many different human cancers. Using quantitative RT-PCR, we have verified a reduction in thyroid cancer and shown, for the first time, that *NDRG2 *mRNA is statistically significantly down-regulated in breast cancer. Furthermore, our observations indicate that other tissues such as cervix and testis can have lower levels of *NDRG2 *mRNA in tumor tissue compared to normal tissue.

## Background

The transformation of normal cells into cancer cells is generally believed to rely on genetic or epigenetic changes in the genome. These changes often activate oncogenes and/or inactivate tumor suppressor genes. The recently described *N-Myc Downstream Regulated Gene 2 *(*NDRG2*) is down-regulated in a variety of human tumors, including colorectal, liver and thyroid cancers as well as glioblastoma [1-5]. Furthermore, elevated expression of *NDRG2 *in tumors correlates with an improved prognosis in gastric cancer, high-grade glioma and hepatocellular carcinomas [2,6,7]. Several *in vitro *studies have demonstrated a reduced cell growth when NDRG2 was over-expressed in cell-lines lacking endogenous expression [5,8]. These data suggest that *NDRG2 *might have a role in suppressing carcinogenesis. *NDRG2 *is one of four members belonging to the *NDRG *family. In contrast to *NDRG1*, however, *NDRG2 *does not appear to be down-regulated by N-myc *in vivo *[9]. All four NDRG family members possess an α/β-hydrolase fold domain but whether or not they have enzymatic activity is presently unclear.

Northern blot analysis was previously used to establish the mRNA expression pattern of *NDRG2 *in normal tissues and it has been shown to have the highest expression in brain, heart, skeletal muscle, kidney and liver and lowest expression in tissues such as colon, spleen, placenta and lung [5,10,11]. How *NDRG2 *is regulated is not well understood, but recently it was shown that Myc, via Miz-1, can repress *NDRG2 *expression [12] and that the Wilms' tumor gene 1 protein as well as p53 can induce expression of *NDRG2 *[13,14]. Furthermore, studies have shown that *NDRG2 *transcription can be subjected to an epigenetic repression through promoter methylation, leading to a decreased expression [15,16]. Finally, NDRG2 protein contains several phosphorylation sites [17] suggesting a completely separate regulatory mechanism via a phosphorylation-dephosphorylation cycle.

Based on our current understanding, *NDRG2 *is a candidate tumor suppressor gene. To further understand how *NDRG2 *is involved in carcinogenesis, we need to know more about the regulation, distribution and function of *NDRG2*. In this paper, we present a broad profile of *NDRG2 *expression in human cancers with focus on breast cancer. Our data from 19 different cancers indicates that *NDRG2 *is up-regulated in around 8% of all tumors examined, unaltered in 62% of all cases and that approximately a third of the tumor samples show down-regulation of *NDRG2 *compared to the corresponding normal tissue. By quantifying the level of *NDRG2 *mRNA in 35 breast cancer samples, we observed a statistically significant down-regulation of *NDRG2 *in tumor samples compared to normal tissue. We also quantified the level of *MYC *in the same breast cancer samples to investigate whether or not a high level of *MYC *expression could be responsible for the observed *NDRG2 *down-regulation. In our hands, we observed a positive correlation, indicating that *MYC *and *NDRG2 *are co-regulated. Thus, another regulatory pathway than repression by Myc appears to reduce *NDRG2 *mRNA levels in breast cancer.

## Methods

### Cancer Profiling Array II

The Cancer Profiling Array II (CPA II, Clontech) was hybridised with 50 ng of radioactively labelled *NDRG2 *probe according to the manufacturer's instructions. The 460 bp *NDRG2 *probe was generated by PCR using the primers 5'CTCACTCTGTGGAGACACCAT3' and 5'GGGTGATATCACCTCCACGCT3'. The hybridised array was exposed to a phosphorimaging screen for 24 hours and the intensity of each spot was quantified using ImageQuant (Molecular Dynamics). The CPA II consists of paired cDNA samples generated from the total RNA of normal and tumor tissue. Because the array is normalised for several housekeeping genes, quantification of the hybridisation signal provides an estimate of relative transcript abundance. Furthermore, the CPA II was hybridised with a human ubiquitin control cDNA probe provided by the manufacturer and exposed for 48 hours. All samples showed uniform expression of ubiquitin confirming the normalisation by the manufacturer (data not shown).

### TissueScan mRNA quantification

Breast and thyroid cancer TissueScan qPCR Arrays (Origene Technologies, Rockville MD) were used to quantify and normalise the level of *NDRG2 *and *MYC *to the expression level of *β-actin*. The TissueScan Arrays consist of pre-normalized cDNA from both normal and tumor tissues. The commercial supplier has confirmed that the removal of human tissue was performed with the approval of an Institutional Review Board ethics committee within each medical center. The medical centers from which the tissue was banked represented major academic medical centers within the US. Tissue and data collection conform to Federal requirements and informed consent was obtained from all human subjects. According to the manufacturer, samples were selected based on tumor content (minimally 50% tumor) as determined by microscopic pathology analysis. Based on a pathology report the tumors were partly classified, following the tumor-node-metastasis (TNM) staging system, according to their size/extent (TI-TIVC). The normal samples were taken from patients diagnosed with cancer, but the tissues were harvested from normal regions. The quantification was performed on an ABI 7300 sequence detector as previously described [1] or as recommended by the manufacturer. The primers and probes for *NDRG2 *have previously been described [1]. Primers and probes for *β-actin *(part no. 4310881) and *MYC *(assay ID Hs00153408 m1) were obtained from Applied Biosystems.

In a validation experiment using a control sample, a 2-fold dilution series was produced and assayed for *NDRG2, MYC *and *β-actin *expression as described in the comparative C_t _method [18]. When C_t _values were plotted against log dilution it was shown that the assays were quantitative over a range of 128-fold dilution for the *NDRG2/β-actin *assay and over a 512-fold dilution for the *MYC/β-actin *assay, using a threshold of 0.2 for *β-actin*, 0.07 for *NDRG2 *and 0.15 for *MYC*.

*NDRG2, MYC *and *β-actin *mRNAs were quantified in separate wells in triplicate. The standard deviation of the same sample (internal and positive control) in separate experiments was 20%, 10% and 20% for *NDRG2, MYC *and *β-actin *respectively in thyroid tissue, and 27%, 10% and 16% for *NDRG2, MYC *and *β-actin *respectively in breast tissue, indicating a day-to-day variation of the assay. Negative controls (without conversion of RNA into cDNA) and positive controls were included in all sets.

For both thyroid and breast cancer samples, some data were excluded either due to C_t _values being too low and outside the standard curve or because the mRNA levels were below the detection level. In general, the levels of both *NDRG2 *and *MYC *were lower in thyroid tissue than breast tissue samples compared to *β-actin*, which was equally expressed in both tissues. The distribution of gender and age among cases whose tissue was included in the final analysis is presented in Tables 1 and 2.

**Table 1:** Characteristics of cases with thyroid gland cancer

	**Normal**^**1**^	TI	TII	TIII	TIVA	TIVC
**n**	4	9	3	6	2	1
**Females**	4	7	2	3	1	-
**Males**	-	2	1	3	1	1
**Mean age**	47.3	40.3	59.3	65.5	48.5	80.0
**SD**	16.3	15.0	3.2	9.4	5.0	-

^1^Normal samples were control tissue taken from patients diagnosed with cancer. SD = standard deviation

**Table 2:** Characteristics of cases with breast cancer

	**Normal**^**1**^	TI	TIIA	TIIB	TIIIA	TIIIC
**n**	7	10	13	7	8	3
**Females**	7	10	13	7	8	3
**Males**	-	-	-	-	-	-
**Mean age**	43.1	63.3	58.2	53.6	55.3	57.7
**SD**	9.6	10.6	13.9	6.0	13.9	9.6

^1^Normal samples were control tissue taken from patients diagnosed with cancer. SD = standard deviation

### Statistical analysis

GraphPad Prism 4 was used for all the statistic calculations and p values < 0.05 were considered significant for all tests. The paired two-tailed t-test was used for comparisons of the CPA data and p-values were subsequently corrected for type I errors using the Benjamini-Hochberg method. For the quantitative RT-PCR data for the breast and thyroid tissues, the Mann-Whitney test was used to compare mean values between normal and tumor samples, since the variances in those samples were different. The non-parametric Kruskal-Wallis test was used on the different tumor stages TI through TIIIC groups, since the variances in the groups were different. For correlation analyses we used Spearman's correlation test due to the number of samples and since the data did not follow a Gaussian distribution.

## Results

### Expression profile of NDRG2 in human cancers

**Figure 1:**
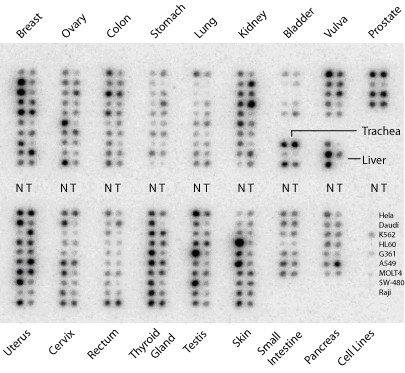
***NDRG2 *mRNA levels in 19 different human tissues composed of paired normal and tumor samples**. The Cancer Profiling Array II was hybridised with a ^32^P-labelled probe for *NDRG2*. Each dot represents one tissue sample and the intensity of each dot was measured with ImageQuant. In 30% of tumor samples, *NDRG2 *mRNA levels were down-regulated by at least 2-fold compared to the corresponding normal tissue. In 8% of tumor samples, *NDRG2 *mRNA levels were increased by 2-fold or more compared to the corresponding normal tissue. N = normal tissue and T = tumor tissue.

By hybridising a CPA with a radioactive labelled probe it was possible to examine the levels of *NDRG2 *mRNA in 19 different human cancers, distributed as 154 paired tissue samples, and 9 different cell lines. Samples on the CPA have been normalised by the manufacturer on the basis of three different housekeeping genes. As shown in Figure 1, *NDRG2 *was detected at varying expression levels in all normal tissues. Dot intensities from the CPA and corresponding patient data are available as supplementary data (Additional file 1), except for colon cancer which was published previously [1]. With regards to normal tissue samples, the highest expression of *NDRG2 *was seen in liver, prostate, breast, uterus, thyroid gland, testis and skin. An intermediate expression level was seen in vulva, trachea, kidney, colon, ovary, cervix, small intestine and pancreas and, finally, lowest expression was observed in bladder, lung, stomach and rectum.

We defined down- and up-regulation as changes in the mRNA level between tumor and corresponding normal tissue to less than or equal to 0.5-fold or more than or equal to 2-fold, respectively. Analysing data from the CPA based on these criteria revealed that 95 out of 154 samples (62%) showed unaltered *NDRG2 *mRNA levels in tumors. However, 46 of the 154 samples analysed (30%) showed down-regulation and in 13 of 154 samples (8%), an up-regulation of the *NDRG2 *mRNA level was observed (Table 3).

**Table 3:** Expression analysis of NDRG2 using a cancer profiling array

Tissue	Number of samples	Paired two-tailed t-test	Down-regulated (Tumor/normal ratio ≤ 0.5)	Unaltered (Tumor/normal ratio between 0.51-1.99)	Up-regulated (Tumor/normal ratio ≥ 2.0)	Benjamini-Hochberg correction
**Down-regulated *NDRG2 *mRNA levels**						

**Liver**	3	NS	3	-	-	NS

**Down-regulated or unaltered *NDRG2 *mRNA levels**						

**Cervix**	10	0.023	3	7	-	NS
**Colon**	10	0.008	4	6	-	NS
**Ovary**	10	NS	3	7	-	NS
**Skin**	10	NS	4	6	-	NS
**Testis**	10	0.030	7	3	-	NS
**Thyroid Gland**	10	0.005	5	5	-	NS
**Vulva**	5	NS	2	3	-	NS

**Unaltered *NDRG2 *mRNA levels**						

**Prostate**	4	NS	-	4	-	NS
**Small Intestine**	7	NS	-	7	-	NS
**Trachea**	3	NS	-	3	-	NS

**Unaltered or up-regulated *NDRG2 *mRNA levels**						

**Bladder**	5	NS	-	3	2	NS
**Stomach**	10	NS	-	8	2	NS

**Down-regulated, unaltered or up-regulated *NDRG2 *mRNA levels**						

**Breast**	10	NS	4	5	1	NS
**Kidney**	10	NS	3	4	3	NS
**Lung**	10	NS	1	8	1	NS
**Pancreas**	7	NS	1	5	1	NS
**Rectum**	10	NS	3	6	1	NS
**Uterus**	10	NS	3	5	2	NS

NS = not significant

The 19 different cancer forms did not all show unidirectional changes in *NDRG2 *mRNA levels (Table 3). Liver cancer showed, as the only tissue, down-regulation in all samples. Tissue samples from cervix, colon, ovary, skin, testis, thyroid gland and vulva all showed both down-regulation and unaltered mRNA levels. Prostate, small intestine and trachea samples were all unaltered, while bladder and stomach had unaltered or up-regulated levels of *NDRG2 *mRNA. Finally, breast, kidney, lung, pancreas, rectum and uterus showed a mixture of down-regulated, unaltered and up-regulated levels.

Calculating a paired two-tailed t-test on our dataset of 154 paired samples, we obtained statistically significant p-values for colon (p = 0.008; n = 10), cervix (p = 0.02; n = 10), thyroid gland (p = 0.005; n = 10) and testis (p = 0.03; n = 10) only. However, after correcting for type I errors, using the Benjamini-Hochberg correction, we found that none of these tissues exhibited significant differences between normal and tumor tissue.

### Levels of NDRG2 and MYC mRNA in thyroid gland cancer

**Figure 2:**
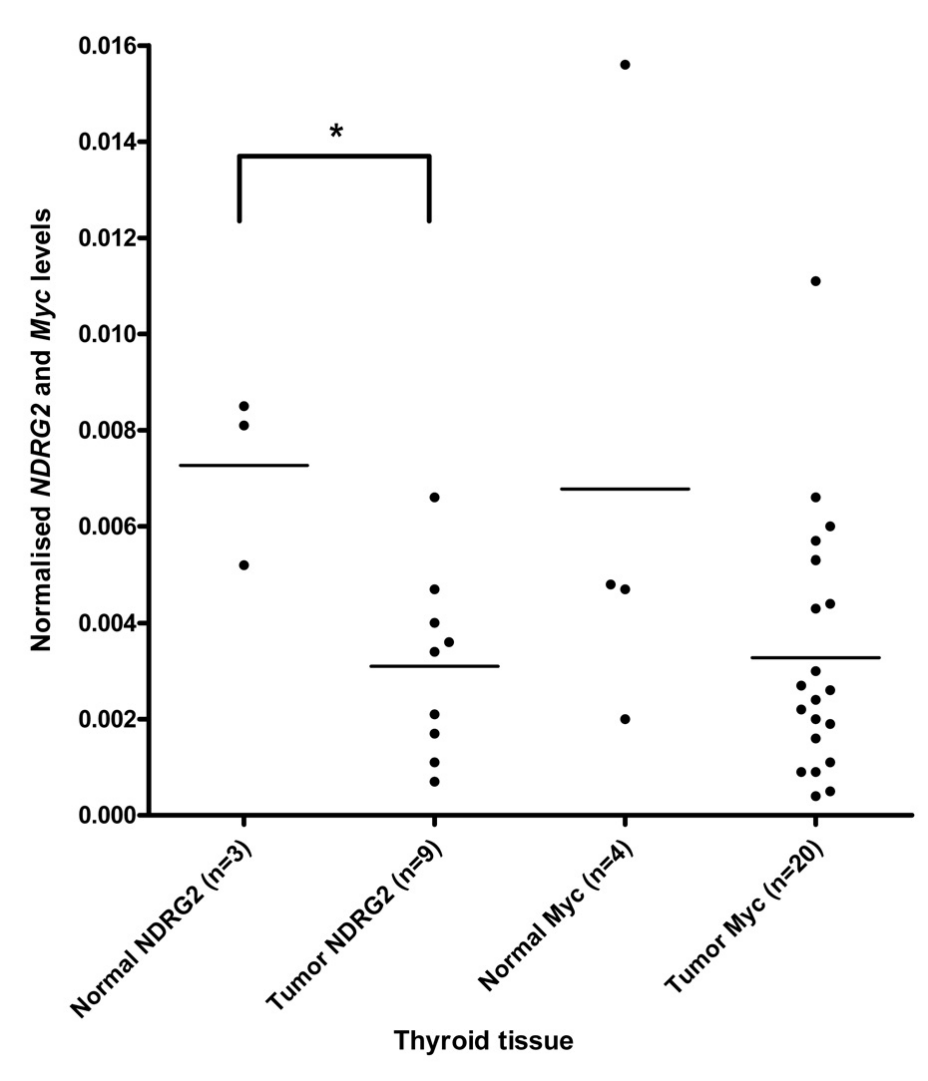
***NDRG2 *mRNA levels are down-regulated in tumor tissue from thyroid cancer patients**. mRNA expression of *NDRG2 *and *Myc*, respectively, was determined by real-time RT-PCR and normalised to *β-actin *in normal and affected tissue from thyroid cancer patients. Lines represent mean expression. * p < 0.05 compared to the normal group using a two-tailed Mann-Whitney test. No correlation was found between *NDRG2 *and *Myc *mRNA levels using a Spearman's correlation test.

To elaborate on some of the data obtained from the CPA, we decided to quantify *NDRG2 *mRNA in sample sets obtained from patients diagnosed with thyroid gland cancer and breast cancer. Both sets included normal and tumor tissues and, furthermore, the tumor samples covered stages TI-TIV. The level of *NDRG2 *mRNA in normal and tumor samples from thyroid gland is shown in Figure 2 and mean values with 95% confidence intervals are presented in Table 4. Using a two-tailed Mann-Whitney test revealed a significant reduction in *NDRG2 *mRNA levels in tumor tissue compared to the corresponding normal tissue (p = 0.02), albeit based on a small sample set.

**Table 4:** Expression values in thyroid gland and breast tissues

	**Mean value in normal tissue **(95% CI)	**Mean value in tumor tissue **(95% CI)
Thyroid gland		
*NDRG2*	0.0073 (0.0028-0.0112)	0.0030 (0.0016-0.0044)
*MYC*	0.0070 (-0.0028-0.0168)	0.0034 (0.0021-0.0046)
**Breast**		
*NDRG2*	0.0256 (0.0063-0.0448)	0.0045 (0.0027-0.0064)
*MYC*	0.0589 (0.0065-0.1112)	0.0282 (0.0177-0.0386)

95% CI = 95% confidence interval

Zhang *et al. *have previously shown that the expression of *NDRG2 *can be repressed by the Myc oncoprotein via Miz-1 [12]. In order to look further into a possible relationship between *NDRG2 *and *MYC *expression, we also quantified the levels of *MYC *mRNA in the same tissue samples that were analysed for *NDRG2 *mRNA levels (Figure 2; Table 4). Examining the level of *MYC *mRNA did not reveal a significant change between normal and cancerous thyroid gland tissues. In order to analyse for a covariation between *NDRG2 *and *MYC*, we calculated the correlation coefficient using a Spearman's correlation test. It was not possible to calculate a correlation coefficient for the normal thyroid gland samples due to too few samples, but for thyroid gland tumors we did not observe a correlation.

### Levels of NDRG2 and MYC mRNA in breast cancer

**Figure 3:**
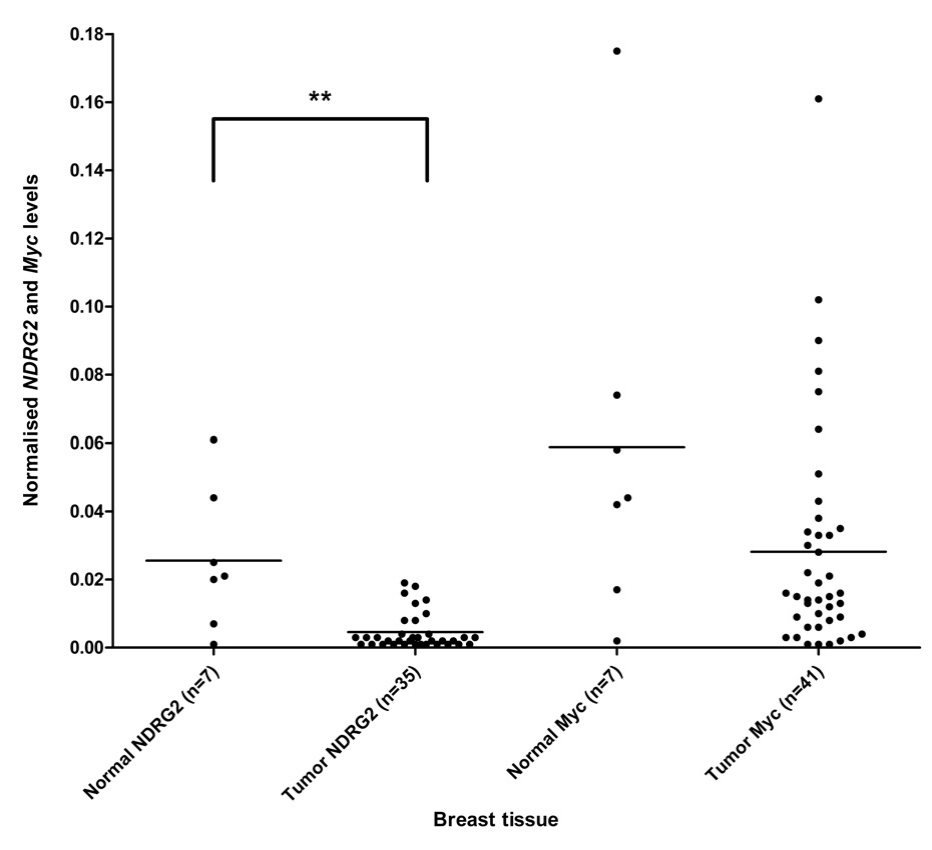
***NDRG2 *mRNA levels are down-regulated in tumor tissue from breast cancer patients and co-vary with *Myc *mRNA levels**. Normalised mRNA expression of *NDRG2 *and *Myc*, respectively, was determined in normal and affected tissue from breast cancer patients. Lines represent mean expression. ** p < 0.005 compared to the normal group using a two-tailed Mann-Whitney test. A weakly significant positive correlation was found between *NDRG2 *and *Myc *mRNA levels for normal samples (p = 0.0238) and for tumor samples (p = 0.0113), using a Spearman's correlation test.

Quantification of *NDRG2 *mRNA in breast cancer tissue showed a clear reduction in the level of *NDRG2 *mRNA in tumors compared to normal tissue (Figure 3, Table 4). The reduction in *NDRG2 *mRNA was statistically significant by a two-tailed Mann-Whitney test (p = 0.004). Classifying tumor samples according to tumor stages TI to TIIIC showed that the different tumor stages did not have significantly different mean values using the Kruskal-Wallis test (p = 0.738, data not shown).

In the same set of breast cancer samples, we quantified the level of *MYC *mRNA (Figure 3; Table 4). Although *MYC *mRNA levels appeared lower in all tumors compared to normal tissue, this was not statistically significant, using a two-tailed Mann-Whitney test (p = 0.0725). Just as for *NDRG2*, we did not observe any significant differences between mean values when analysing tumors from stages TI - TIIIC (Kruskal-Wallis test, p = 0.180 and data not shown).

We used a Spearman's correlation test in order to analyse for a possible relationship between *NDRG2 *and *MYC *expression. For breast cancer we obtained a statistically significant, but weak, correlation coefficient of r = 0.8571 (p = 0.0238) for normal samples and r = 0.4234 (p = 0.0113) for tumor samples. A positive correlation coefficient between 0-1 indicates that the two variables being analysed either decrease or increase together.

## Discussion

In order to understand the initiation and progression of cancer, it is important to identify the genes and mechanisms involved. In this paper, we have examined the expression of *NDRG2*, a recently discovered gene with a proposed tumor suppressor activity, using a commercially available cancer profiling array covering 19 different human cancer forms. One third of all tumor samples analysed showed down-regulation of *NDRG2 *mRNA levels by 2-fold or more compared to corresponding normal tissue, suggesting that either the regulators of *NDRG2 *are generally affected or that *NDRG2 *is often inactivated in tumors. Several tumor types (cervix, colon, testis and thyroid) from the CPA showed a statistically significant reduction of *NDRG2 *mRNA when analysed separately (t-test), but when corrected together for type I errors (Benjamini-Hochberg correction) covering the whole array, none of them retained significance. However, analysing both colon and thyroid cancer in a more quantitative manner and using larger sample sets, we and others have demonstrated a clear and significant decrease in *NDRG2 *mRNA levels in both cancer types [1,3]. Similarly, we observed lower levels in all 3 liver cancer samples, which was recently confirmed in a larger study [2]. From the CPA results, we would also like to highlight tissues such as cervix and testis as candidate tissues with a potential decrease of *NDRG2 *mRNA between normal and tumor tissues. The sample size for each cancer type on the CPA was between 3 to 10 paired normal and tumor samples. A conclusion from the CPA is therefore not straightforward and we only used the array data as a survey-type study to identify candidate tissues for subsequent quantification studies.

In the case of both breast and thyroid cancer, we analysed 5-7 normal and around 40 tumor samples by quantitative RT-PCR. All samples showed a uniform level of β-actin mRNA, indicating equivalent amounts of mRNA in each sample. However, in some cases the *NDRG2 *levels were too low (outside the standard curve) or even undetectable. In the case of thyroid cancer, 2 normal and the majority of tumor samples (34) were discarded due to too low or undetectable mRNA levels. Thus, our statistical analysis was based on 3 normal and 9 tumor samples. Although we observed a significant difference between normal and tumor tissues with regard to *NDRG2 *mRNA levels, we are aware that it is not a satisfying sample size to draw any conclusions from, but as mentioned previously our data are consistent with recent findings by others [3].

Using quantitative RT-PCR, we demonstrated that *NDRG2 *mRNA levels were statistically significantly reduced in breast cancer tissue when compared to normal tissue. Liu *et al. *have previously reported by semi-quantitative PCR that there was a reduction in *NDRG2 *mRNA levels in 5 out of 21 breast cancer samples tested, compared to normal tissue [16]. However, our results are based on quantitative real-time PCR and a slightly larger sample set (n = 35). Differences at the mRNA level for *NDRG2 *are likely to be reflected at the protein level [12] and our results are in agreement with other studies showing that NDRG2 protein is reduced in breast cancer [19].

Two alternative protein isoforms of NDRG2 have been described. In the long form of NDRG2, a short stretch of 14 amino acids is inserted N-terminal to the conserved α/β-hydrolase fold domain, as a result of alternative splicing [11,20]. It would be interesting in future studies to examine whether the different splice forms of *NDRG2 *are under differential regulation, which could explain the mixed expression results observed in some cancer types and shed light on the possible function of this protein.

In 2006 it was shown that the Myc oncoprotein could interact with the *NDRG2 *promoter via Miz-1 and that the level of Myc is inversely correlated with the level of NDRG2 in colon cancer cell-lines undergoing differentiation [12]. Since *MYC *is known to be either over-expressed or amplified in many human cancers [21], it could be responsible for the decreased levels of *NDRG2 *mRNA. Zhao *et al. *demonstrated that there is an inverse correlation between the levels of *NDRG2 *and *MYC *mRNA in thyroid cancer [3]. Whether this also applies to other cancers is not known, and we therefore quantified *MYC *and *NDRG2 *mRNA in a set of breast cancer samples. However, we observed only a weak correlation between *MYC *and *NDRG2 *mRNA expression in normal and tumor samples from breast tissue, indicating that *MYC *and *NDRG2 *are co-regulated. This makes it unlikely that Myc acts as a repressor for *NDRG2 *gene expression in breast cancer. One possibility is that Myc acts as a transcriptional activator for *NDRG2 *by recruiting co-factors other than Miz-1 [22]. Alternatively, the observed correlation of *MYC *and *NDRG2 *mRNA expression in breast tissue may result from a shared regulatory mechanism of these genes in the normal and tumor samples tested.

In summary, we have demonstrated that *NDRG2 *mRNA levels are significantly reduced in thyroid and breast cancer, and that *NDRG2 *mRNA levels will be interesting to quantify in cervix and testis cancer. Overexpression of NDRG2 protein in cancer cell-lines results in a marked reduction in cell proliferation, although the precise mechanisms are unclear [5,8,23]. Since studies in other cancer types demonstrate that high *NDRG2 *mRNA expression correlates with improved prognosis [2,6,7], future studies could be aimed at developing therapeutic approaches to increase NDRG2 expression in tumor tissue.

## Conclusion

In conclusion, *NDRG2 *mRNA levels were significantly decreased in tumor samples from both thyroid and breast cancer, compared to normal tissue. Our observations also indicate that cervix and testis tissues might exhibit decreases in *NDRG2 *mRNA in a similar manner. Finally, our results suggest that the observed reduction of *NDRG2 *mRNA in breast cancer correlated weakly with *MYC *mRNA expression. Although expression of *NDRG2 *mRNA is reduced in many human tumors, the role of *NDRG2 *in carcinogenesis is presently unclear.

## Competing interests

The authors declare that they have no competing interests.

## Authors' contributions

CM and AL conceived the idea of the study. RL and AL carried out the Cancer Profiling Array analysis. AL made all the quantitative RT-PCR, supervised by JB. CM and AL drafted the manuscript. LV and JB contributed to the manuscript and gave statistical advice. All authors contributed to interpretation and discussion of the results and read and approved the final version.

## Pre-publication history

The pre-publication history for this paper can be accessed here:

http://www.biomedcentral.com/1471-2407/11/14/prepub

## Supplementary Material

Additional file 1 **Expression analysis of NDRG2 using a cancer profiling array**. Signal intensities from the CPA and corresponding patient data. Data for colon cancer was published previously [1]. Click here for file
