# Supplementary material for: Expression profile of the N-myc Downstream Regulated Gene 2 (NDRG2) in human cancers with focus on breast cancer
Source: BMC Cancer. 2011 Jan 12;11:14. doi: 10.1186/1471-2407-11-14 (PMC3024299; doi:10.1186/1471-2407-11-14)
Supplement: Additional file 1 — Expression analysis of NDRG2 using a cancer profiling array. Signal intensities from the CPA and corresponding patient data. Data for colon cancer was published previously [1]. [file 1471-2407-11-14-S1.DOC]

**Additional file 1**

The TNM staging system describes the extent of the primary tumor (T), the absence/presence of metastasis to nearby lymph nodes (N) and the absence/presence of distant metastases (M). N/A: Information Not Available

**Table S1 Expression analysis of *NDRG2* in** Prostate Cancer

| **Sample** | **Signal intensity** | **Tumor/normal** | **TNM staging** | **Age** | **Gender** |
| --- | --- | --- | --- | --- | --- |
| Tumor/ normal | 95064/ 94949 | 1.00 | I; T1N0M0 | 62 | Male |
| Tumor/ normal | 18216/ 26245 | 0.69 | N/A; N/A | 65 | Male |
| Tumor/ normal | 98668/ 77705 | 1.27 | N/A; N/A | 67 | Male |
| Tumor/ normal | 68972/ 65151 | 1.06 | N/A; N/A | 68 | Male |

**Table S2 Expression analysis of *NDRG2* in** Vulva Cancer

| **Sample** | **Signal intensity** | **Tumor/normal** | **TNM staging** | **Age** | **Gender** |
| --- | --- | --- | --- | --- | --- |
| Tumor/ normal | 59822/ 168745 | 0.35 | II; T2N0M0 | 80 | Female |
| Tumor/ normal | 58802/ 63282 | 0.93 | II; T2N0M0 | 78 | Female |
| Tumor/ normal | 78086/ 47264 | 1.65 | II; T2N0M0 | 78 | Female |
| Tumor/ normal | 18750/ 43616 | 0.43 | II; T2N0M0 | 74 | Female |
| Tumor/ normal | 23542/ 22460 | 1.05 | N/A; T2aN1M0 | 60 | Female |

**Table S3 Expression analysis of *NDRG2* in** **Bladder Cancer**

| **Sample** | **Signal intensity** | **Tumor/normal** | **TNM staging** | **Age** | **Gender** |
| --- | --- | --- | --- | --- | --- |
| Tumor/ normal | 12599/ 15995 | 0.79 | II; T2N0M0 | 60 | Male |
| Tumor/ normal | 11088/ 5231 | 2.12 | III; T3N0M0 | 54 | Male |
| Tumor/ normal | 3835/ 2993 | 1.28 | IV; T4bN0M0 | 62 | Male |
| Tumor/ normal | 11814/ 7415 | 1.59 | II; T2N0M0 | 59 | Female |
| Tumor/ normal | 29699/ 12043 | 2.47 | III; T3aN0M0 | 56 | Male |

**Table S4 Expression analysis of *NDRG2* in** **Liver Cancer**

| **Sample** | **Signal intensity** | **Tumor/normal** | **TNM staging** | **Age** | **Gender** |
| --- | --- | --- | --- | --- | --- |
| Tumor/ normal | 7169/ 49543 | 0.14 | N/A; T3N0M0 | 59 | Male |
| Tumor/ normal | 44805/ 200355 | 0.22 | N/A; T2N0M0 | 36 | Male |
| Tumor/ normal | 7425/ 109411 | 0.07 | N/A; T2N0M0 | 58 | Male |

**Table S5 Expression analysis of *NDRG2* in** **Trachea Cancer**

| **Sample** | **Signal intensity** | **Tumor/normal** | **TNM staging** | **Age** | **Gender** |
| --- | --- | --- | --- | --- | --- |
| Tumor/ normal | 119937/ 86472 | 1.39 | N/A; T4N1M0 | 44 | Female |
| Tumor/ normal | 10059/ 17786 | 0.57 | N/A; T4N0M0 | 54 | Female |
| Tumor/ normal | 41946/ 77710 | 0.54 | N/A; T3N1M0 | 54 | Male |

**Table S6 Expression analysis of *NDRG2* in** **Kidney Cancer**

| **Sample** | **Signal intensity** | **Tumor/normal** | **TNM staging** | **Age** | **Gender** |
| --- | --- | --- | --- | --- | --- |
| Tumor/ normal | 20860/ 40727 | 0.51 | II; T2N0M0 | 59 | Male |
| Tumor/ normal | 115455/ 37668 | 3.07 | N/A; N/A | 60 | Male |
| Tumor/ normal | 63568/ 61953 | 1.03 | IV; T3aN0M1 | 69 | Female |
| Tumor/ normal | 223980/ 79990 | 2.80 | III; T3aN0M0 | 55 | Male |
| Tumor/ normal | 21164/ 63161 | 0.34 | III; T3bN0M0 | 58 | Male |
| Tumor/ normal | 38139/ 82578 | 0.46 | III; T3aN0M0 | 41 | Male |
| Tumor/ normal | 23786/ 40767 | 0.58 | III; T2N1M0 | 79 | Male |
| Tumor/ normal | 12176/ 41291 | 0.29 | IV; T3bN1M0 | 69 | Female |
| Tumor/ normal | 55598/ 28472 | 1.95 | IIB; T3N0M0 | 22 | Female |
| Tumor/ normal | 32293/ 10008 | 3.23 | N/A; T3aNxM0 | 58 | Male |

**Table S7 Expression analysis of *NDRG2* in** **Lung Cancer**

| **Sample** | **Signal intensity** | **Tumor/normal** | **TNM staging** | **Age** | **Gender** |
| --- | --- | --- | --- | --- | --- |
| Tumor/ normal | 18902/ 43838 | 0.43 | II; T2N1M0 | 69 | Female |
| Tumor/ normal | 5216/ 7596 | 0.69 | N/A; T3N2M0 | 72 | Male |
| Tumor/ normal | 4288/ 5568 | 0.77 | N/A; T3N1M0 | 66 | Male |
| Tumor/ normal | 16450/ 7834 | 2.10 | N/A; T3N0M0 | 51 | Male |
| Tumor/ normal | 15921/ 8689 | 1.83 | IB; T2N0M0 | 63 | Male |
| Tumor/ normal | 28679/ 19683 | 1.46 | I; T2N0M0 | 62 | Male |
| Tumor/ normal | 7811/ 12623 | 0.62 | IIIA; T3N1M0 | 67 | Male |
| Tumor/ normal | 15099/ 12160 | 1.24 | IIB; T2N1M0 | 71 | Male |
| Tumor/ normal | 15833/ 26154 | 0.61 | N/A; N/A | 65 | Male |
| Tumor/ normal | 29405/ 26732 | 1.10 | IB; T2N0M0 | 52 | Male |

**Table S8 Expression analysis of *NDRG2* in Stomach Cancer**

| **Sample** | **Signal intensity** | **Tumor/normal** | **TNM staging** | **Age** | **Gender** |
| --- | --- | --- | --- | --- | --- |
| Tumor/ normal | 26211/ 13045 | 2.01 | IIB; T3N0M0 | 63 | Male |
| Tumor/ normal | 6433/ 11243 | 0.57 | N/A; T3N1Mx | 57 | Male |
| Tumor/ normal | 19474/ 10827 | 1.80 | N/A; T3N0M0 | 47 | Male |
| Tumor/ normal | 44788/ 9019 | 4.97 | IB; T2N0M0 | 65 | Male |
| Tumor/ normal | 21788/ 16035 | 1.36 | IB; T2N0M0 | 61 | Male |
| Tumor/ normal | 14812/ 9114 | 1.63 | IV; T4N3M0 | 50 | Male |
| Tumor/ normal | 22773/ 21642 | 1.05 | IV; T4N2M0 | 72 | Female |
| Tumor/ normal | 7663/ 6859 | 1.12 | IIIA; T3N1M0 | 67 | Female |
| Tumor/ normal | 18015/ 33679 | 0.53 | IIIA; T3N1M0 | 75 | Male |
| Tumor/ normal | 4907/ 6934 | 0.71 | III; T4N0M0 | 68 | Female |

**Table S9 Expression analysis of *NDRG2* in Ovary Cancer**

| **Sample** | **Signal intensity** | **Tumor/normal** | **TNM staging** | **Age** | **Gender** |
| --- | --- | --- | --- | --- | --- |
| Tumor/ normal | 12114/ 34045 | 0.36 | IA; T1a2N0M0 | 38 | Female |
| Tumor/ normal | 33236/ 43134 | 0.77 | N/A; N/A | 53 | Female |
| Tumor/ normal | 57606/ 31672 | 1.82 | III; T3NxM0 | 61 | Female |
| Tumor/ normal | 19973/ 31525 | 0.64 | IIIC; T3cN1M0 | 38 | Female |
| Tumor/ normal | 25473/ 29507 | 0.86 | N/A; T2NxM0 | 55 | Female |
| Tumor/ normal | 12975/ 134780 | 0.10 | IIC; T2cN0M0 | 54 | Female |
| Tumor/ normal | 48788/ 44083 | 1.11 | IA; N/A | 63 | Female |
| Tumor/ normal | 22652/ 31363 | 0.72 | IVB; N/A | 72 | Female |
| Tumor/ normal | 14342/ 26670 | 0.54 | IIIC; T3N0M0 | 36 | Female |
| Tumor/ normal | 23528/ 101745 | 0.23 | IIIA; T3NxM0 | 53 | Female |

**Table S10 Expression analysis of *NDRG2* in Breast Cancer**

| **Sample** | **Signal intensity** | **Tumor/normal** | **TNM staging** | **Age** | **Gender** |
| --- | --- | --- | --- | --- | --- |
| Tumor/ normal | 26122/ 31985 | 0.81 | I; N/A | 59 | Female |
| Tumor/ normal | 36128/ 246633 | 0.15 | IIIA; T1N2M0 | 60 | Female |
| Tumor/ normal | 31062/ 260463 | 0.12 | I; T1N0M0 | 53 | Female |
| Tumor/ normal | 59685/ 93221 | 0.64 | IIIA; T3N1M0 | 42 | Female |
| Tumor/ normal | 90912/ 151000 | 0.60 | II; T2N0M0 | 56 | Female |
| Tumor/ normal | 15527/ 45584 | 0.34 | IIIB; T4N1M0 | 52 | Female |
| Tumor/ normal | 45689/ 38097 | 1.20 | IIB; T2N1M0 | 71 | Female |
| Tumor/ normal | 16399/ 74227 | 0.22 | IIB, T2N1M0 | 48 | Female |
| Tumor/ normal | 110706/ 55259 | 2.00 | I; T1N0M0 | 46 | Female |
| Tumor/ normal | 40134/ 41779 | 0.96 | IIB; T2N1M0 | 45 | Female |

**Table S11 Expression analysis of *NDRG2* in Uterus Cancer**

| **Sample** | **Signal intensity** | **Tumor/normal** | **TNM staging** | **Age** | **Gender** |
| --- | --- | --- | --- | --- | --- |
| Tumor/ normal | 106160/ 94637 | 1.12 | I; T1N0M0 | 50 | Female |
| Tumor/ normal | 42585/ 132522 | 0.32 | I; T1b1N0M0 | 75 | Female |
| Tumor/ normal | 98607/ 20080 | 4.91 | N/A; N/A | 77 | Female |
| Tumor/ normal | 73169/ 75596 | 0.97 | N/A; N/A | 46 | Female |
| Tumor/ normal | 53782/ 46404 | 1.16 | N/A; N/A | 55 | Female |
| Tumor/ normal | 147938/ 73231 | 2.02 | IIB; T2N0M0 | 46 | Female |
| Tumor/ normal | 97924/ 109044 | 0.90 | IA; T1aN0M0 | 43 | Female |
| Tumor/ normal | 32383/ 155829 | 0.21 | IB; T1b1N0M0 | 40 | Female |
| Tumor/ normal | 26942/ 23647 | 1.14 | IIB; T3N0M0 | 71 | Female |
| Tumor/ normal | 28922/ 88329 | 0.33 | I; T1cN0M0 | 69 | Female |

**Table S12 Expression analysis of *NDRG2* in Cervix Cancer**

| **Sample** | **Signal intensity** | **Tumor/normal** | **TNM staging** | **Age** | **Gender** |
| --- | --- | --- | --- | --- | --- |
| Tumor/ normal | 51269/ 45337 | 1.13 | IIIB; T1b1N1M0 | 52 | Female |
| Tumor/ normal | 12754/ 73717 | 0.17 | IIA; T2N0M0 | 69 | Female |
| Tumor/ normal | 8399/ 12494 | 0.67 | IIIB; T1b2N1M0 | 36 | Female |
| Tumor/ normal | 17103/ 19917 | 0.86 | IIA; T2aN0M0 | 52 | Female |
| Tumor/ normal | 10364/ 18484 | 0.56 | II; T2aN0M0 | 63 | Female |
| Tumor/ normal | 46755/ 66084 | 0.71 | IB2; T1b2N0M0 | 50 | Female |
| Tumor/ normal | 16573/ 63935 | 0.26 | IA1; T1a1N0M0 | 50 | Female |
| Tumor/ normal | 16534/ 28151 | 0.59 | IB1; T1b1N0M0 | 56 | Female |
| Tumor/ normal | 19664/ 49809 | 0.39 | IB2; T1b2N0M0 | 26 | Female |
| Tumor/ normal | 45858/ 53090 | 0.86 | IB; T1b1N0M0 | 44 | Female |

**Table S13 Expression analysis of *NDRG2* in Rectum Cancer**

| **Sample** | **Signal intensity** | **Tumor/normal** | **TNM staging** | **Age** | **Gender** |
| --- | --- | --- | --- | --- | --- |
| Tumor/ normal | 14378/ 37425 | 0.38 | IVB; T4N2M1 | 49 | Female |
| Tumor/ normal | 36974/ 16300 | 2.27 | IIIA; T4N0M0 | 59 | Female |
| Tumor/ normal | 7577/ 21860 | 0.35 | IV; T4N2M1 | 53 | Female |
| Tumor/ normal | 10430/ 10046 | 1.04 | IIIB; T3N2M0 | 70 | Female |
| Tumor/ normal | 8735/ 14251 | 0.61 | III; T3N1M0 | 42 | Male |
| Tumor/ normal | 10925/ 13379 | 0.82 | II; T3N0M0 | 71 | Female |
| Tumor/ normal | 18690/ 21980 | 0.85 | II; T3N0M0 | 59 | Female |
| Tumor/ normal | 6742/ 18246 | 0.37 | II; T3N0M0 | 43 | Male |
| Tumor/ normal | 14922/ 18812 | 0.79 | IIIB; T4N1M0 | 44 | Female |
| Tumor/ normal | 63366/ 54808 | 1.16 | III; T3N1M0 | 68 | Male |

**Table S14 Expression analysis of *NDRG2* in Thyroid Gland Cancer**

| **Sample** | **Signal intensity** | **Tumor/normal** | **TNM staging** | **Age** | **Gender** |
| --- | --- | --- | --- | --- | --- |
| Tumor/ normal | 32596/ 65428 | 0.50 | I; T3N1M0 | 29 | Female |
| Tumor/ normal | 9472/ 81293 | 0.12 | IV; T4N1M0 | 66 | Male |
| Tumor/ normal | 61050/ 87100 | 0.70 | N/A; T3N0M0 | 49 | Female |
| Tumor/ normal | 51550/ 66773 | 0.77 | II; T3N0M0 | 62 | Female |
| Tumor/ normal | 22614/ 127501 | 0.18 | III; T4N1aM0 | 69 | Female |
| Tumor/ normal | 59172/ 43335 | 1.37 | II; T3N0M0 | 70 | Female |
| Tumor/ normal | 51837/ 62300 | 0.83 | II; T2N0M0 | 54 | Female |
| Tumor/ normal | 19057/ 82797 | 0.23 | I; T4N1bM0 | 22 | Female |
| Tumor/ normal | 31578/ 106195 | 0.30 | II; T2N0M0 | 49 | Female |
| Tumor/ normal | 61932/ 97501 | 0.64 | II; T2N0M0 | 63 | Female |

**Table S15 Expression analysis of *NDRG2* in Testis Cancer**

| **Sample** | **Signal intensity** | **Tumor/normal** | **TNM staging** | **Age** | **Gender** |
| --- | --- | --- | --- | --- | --- |
| Tumor/ normal | 46098/ 140190 | 0.33 | I; T2N0M0 | 37 | Male |
| Tumor/ normal | 14404/ 53853 | 0.27 | II; T2N0M0 | 45 | Male |
| Tumor/ normal | 28465/ 34779 | 0.82 | I; T1N0M0 | 21 | Male |
| Tumor/ normal | 59024/ 73936 | 0.80 | I; T1N0M0 | 32 | Male |
| Tumor/ normal | 24055/ 277496 | 0.09 | I; T1N0M0 | 26 | Male |
| Tumor/ normal | 19000/ 50069 | 0.38 | I; T1N0M0 | 18 | Male |
| Tumor/ normal | 39813/ 54927 | 0.72 | IV; T2NxM1 | 22 | Male |
| Tumor/ normal | 20239/ 106963 | 0.19 | I; T1N0M0 | 32 | Male |
| Tumor/ normal | 15663/ 52453 | 0.30 | IV; T1N3M1 | 38 | Male |
| Tumor/ normal | 24333/ 49521 | 0.49 | III; T3N2M0 | 28 | Male |

**Table S16 Expression analysis of *NDRG2* in Skin Cancer**

| **Sample** | **Signal intensity** | **Tumor/normal** | **TNM staging** | **Age** | **Gender** |
| --- | --- | --- | --- | --- | --- |
| Tumor/ normal | 20647/ 10673 | 1.93 | I; T2N0M0 | 49 | Female |
| Tumor/ normal | 22482/ 16890 | 1.33 | III; T4N0M0 | 45 | Female |
| Tumor/ normal | 19876/ 38769 | 0.51 | III; T4N0M0 | 73 | Male |
| Tumor/ normal | 26788/ 449040 | 0.06 | IV; N/A | 58 | Male |
| Tumor/ normal | 26385/ 98198 | 0.27 | III; T4N0M0 | 74 | Female |
| Tumor/ normal | 29010/ 197864 | 0.15 | N/A; N/A | 64 | Male |
| Tumor/ normal | 48754/ 60084 | 0.81 | II; T2N0M0 | 70 | Female |
| Tumor/ normal | 43087/ 47570 | 0.91 | II; T2N0M0 | 68 | Male |
| Tumor/ normal | 38234/ 57209 | 0.67 | III; T4N0M0 | 40 | Female |
| Tumor/ normal | 33888/ 74496 | 0.45 | I; T2N0M0 | 43 | Female |

**Table S17 Expression analysis of *NDRG2* in Small Intestine Cancer**

| **Sample** | **Signal intensity** | **Tumor/normal** | **TNM staging** | **Age** | **Gender** |
| --- | --- | --- | --- | --- | --- |
| Tumor/ normal | 31452/ 36418 | 0.86 | III; T1N1M0 | 49 | Female |
| Tumor/ normal | 35569/ 40481 | 0.88 | II; T3N0M0 | 46 | Male |
| Tumor/ normal | 27158/ 19081 | 1.42 | II; T3N0M0 | 65 | Female |
| Tumor/ normal | 9672/ 8111 | 1.19 | II; T2N0M0 | 63 | Female |
| Tumor/ normal | 20130/ 30736 | 0.65 | N/A; N/A | 59 | Male |
| Tumor/ normal | 30596/ 56854 | 0.54 | III; T3N1M0 | 70 | Female |
| Tumor/ normal | 29819/ 45242 | 0.66 | IV; T4N1M1 | 52 | Male |

**Table S18 Expression analysis of *NDRG2* in Pancreas Cancer**

| **Sample** | **Signal intensity** | **Tumor/normal** | **TNM staging** | **Age** | **Gender** |
| --- | --- | --- | --- | --- | --- |
| Tumor/ normal | 14467/ 45257 | 0.32 | I; N/A | 56 | Female |
| Tumor/ normal | 27771/ 28464 | 0.98 | I; T2N0M0 | 63 | Female |
| Tumor/ normal | 28422/ 36015 | 0.79 | III; T2N1M0 | 40 | Male |
| Tumor/ normal | 25940/ 40673 | 0.64 | II; T3N0M0 | 65 | Male |
| Tumor/ normal | 15379/ 23007 | 0.67 | IVA; T3N1M1 | 51 | Female |
| Tumor/ normal | 87402/ 32634 | 2.68 | N/A; N/A | 53 | Female |
| Tumor/ normal | 20218/ 13567 | 1.48 | N/A; T3NxM0 | 40 | Male |
